# Construction of CNC@SiO_2_@PL Based Superhydrophobic Wood with Excellent Abrasion Resistance Based on Nanoindentation Analysis and Good UV Resistance

**DOI:** 10.3390/polym15040933

**Published:** 2023-02-13

**Authors:** Zhupeng Zhang, Changying Ren, Yi Sun, Yu Miao, Lan Deng, Zepeng Wang, Yizhong Cao, Wenbiao Zhang, Jingda Huang

**Affiliations:** 1 College of Chemistry and Materials Engineering, Zhejiang A&F University, Hangzhou 311300, China; 2 Shandong Institute for Product Quality Inspection, Jinan 250102, China

**Keywords:** CNC, SiO_2_, lignin, superhydrophobic, nanoindentation

## Abstract

Construction of superhydrophobic woods with high abrasion resistance is still a major challenge, and micro analysis for abrasion resistance is scarce. To improve these issues, cellulose nanocrystals (CNC)@SiO_2_@phosphorylated lignin (PL) rods were prepared by SiO_2_ in situ generated on CNC, and then the modified lignin attached to the CNC@SiO_2_ rods surface. Subsequently, the superhydrophobic coating was constructed using hydrophobic modified CNC@SiO_2_@PL rods as the main structural substance by simple spraying or rolling them onto wood surfaces, and both polydimethylsiloxane (PDMS) and epoxy resin were used as the adhesives. The resulting coating had excellent superhydrophobic properties with a water contact angle (WCA) of 157.4° and a slide angle (SA) of 6°. The introduced PL could enhance ultraviolet (UV) resistance of the coating due to the presence of these groups that absorbed UV light in lignin. In the abrasion resistance test, compared with the SiO_2_/PL coating, the abrasion resistance of the one with CNC was much higher, suggesting that CNC could improve the abrasion resistance of the coating due to its high crystallinity and excellent mechanical strength. The coating with PDMS performed better than the one with epoxy resin because the soft surface could offset part of the external impact by deformation in the abrasion process. This was also consistent with the results of the nanoindentation (NI) tests. In view of the simple preparation and good performance, this superhydrophobic wood will have broad application potential.

## 1. Introduction

Lignin is an abundant and renewable material and a byproduct of the forestry chemical industry, but it presently has low application value [1]. If treated improperly, it will even cause some environmental pollution [2]. In recent years, research on the application and development of high-value lignin has been in full swing. Research has shown huge potential applications in drug loading, electrochemistry, catalysis, mildew prevention, and other fields [3]. In particular, the successful development of lignin nanospheres played a positive role in expanding the application range of lignin [4]. Lignin itself contains large numbers of ultraviolet (UV) absorbing groups such as benzene rings, carbonyl, double bonds, and other conjugated structures, which have good UV resistance effects [5]. This also lays a foundation for its application in outdoor materials.

Superhydrophobic surfaces with a water contact angle (WCA) > 150° and a slide angle (SA) < 10° show outstanding properties such as self-cleaning, antifouling, ice resistance, drag reduction, oil-water separation, etc. [6,7,8,9], which could have potential applications in various fields and have become one of the hot research spots over the past 30 years. It is well known that the two basic conditions for reaching superhydrophobicity are both suitable micro–nano layered structure and low surface free energy [10]. There are many ways to construct superhydrophobic surfaces. The diversified preparation means, including chemical vapor deposition (CVD) [11], etching, sol-gel [12], self-assembly [13], impression, spraying [14], etc., endowed superhydrophobic surfaces’ rapid development. However, some methods were limited to high-cost equipment, poor environmental processes, and tedious steps, etc., which were difficult to use at scale. Spraying was the most common and convenient way in line with the large-scale application, which was not limited to the shape and size of the sample, etc. To achieve spraying, it is necessary to allow the corresponding material to disperse into the solvent to form a paint. Currently, inorganic nanoparticles were the common substances used to construct superhydrophobic coatings due to their good stability and economic applicability [15]. Although most inorganic superhydrophobic coatings had good stability, their durability was generally relatively poor and needed further improvement. The inorganic superhydrophobic coating binding with lignin was one of the possible methods to improve durability [16].

Besides durability, the mechanical strength of superhydrophobic coatings was thought one of the most difficult problems to solve. Because the superhydrophobic surface was needed to build the micro–nano rough structure, the mechanical stability of such a structure was insufficient to withstand destruction from various external forces [17]. Many methods were proposed to improve it. For example, by using high-strength adhesives, it could estimate the adhesion between the coating and the substrate, but the stability of the micro–nano structure of the coating was also difficult to solve [18]. The mechanical strength of the rough structure formed by the mutual stacking of particles was unstable enough due to the lack of sufficient binding force among particles [6]. Therefore, it is crucial to enhance the stability between the particles of the microstructure [19]. Moreover, the introduction of naturally degradable organic materials to prepare organic–inorganic composite superhydrophobic coatings was conducive to reducing the burden on the environment. The addition of natural polymers might enhance the performance of the coating or introduce other functions. CNC is a rod-like structure material, which is mainly made of plant fibers by acid hydrolysis, and it has negatively charged sulfonic acid groups on its surface. It has emerged as a strengthening material in recent years [20,21]. As in our previous study [6], we proposed using the CNC@SiO_2_ rods instead of pure SiO_2_ nanoparticles to build the rough structure of the superhydrophobic coating. Compared with the pure SiO_2_ coating, the resulting coating showed ultra-high mechanical abrasion resistance. However, its preparation was performed by a two-step process and needed to use a fluorinated reagent during modification in ethanol solvent, which needs to be improved in terms of simplicity, aging resistance, and environmental protection.

Here, to improve the above problems, the superhydrophobic coating with a fluorine-free reagent was prepared with a one-step spray. The phosphorylated lignin (PL) was introduced onto the CNC@SiO_2_ rods to improve its aging resistance and to enhance its durability [22], as well as be conducive to the dispersion of the rods due to the negative charge on the surface of phosphorylated lignin [23]. Wood was chosen as the substrate due to its strong hydrophility, which is easily affected by the humidity of the surrounding air, showing poor dimensional stability. As well as with the long-time radiation of UV light on wood, there will be the formation of free radicals on the wood surface. The molecular chain of cellulose and hemicellulose is easily destroyed by free radicals, reducing the stability and durability of wood [24]. Therefore, it needs to be modified as hydrophobic and improve its aging resistance to enhance its dimensional stability and UV resistance. It is also difficult to accurately evaluate the mechanical properties of superhydrophobic coatings only by sandpaper abrasion. Nanoindentation technology is generally used to analyze the bond strength of the interface and to receive feedback on the strength region of the interface through the change of the probe mechanical curve [25]. Here, we also tried to evaluate the mechanical strength of superhydrophobic coatings based on the nanomechanics method, which reflected the strength of the constructed micro–nano structures.

## 2. Eexperimental Section

### 2.1. Materials

Enzymatic hydrolysis lignin raw material (content of about 20%) was purchased from Hong Kong Laihe Biotechnology Co., Ltd. Cellulose nanocrystals (CNC) aqueous suspension with a concentration of 12% was purchased from the process development center, the University of Maine, USA. Tetraethyl orthosilicate (TEOS, 98%), ammonium hydroxide (25%), anhydrous ethanol (99%), and sodium phosphate dibasic dodecahydrate (Na_2_HPO_4_∙12H_2_O, white crystalline powder) were purchased from Macklin, China. Both methyltrimethoxysilane (MTMS, C_4_H_12_O_3_Si, Mw~136.22, 98%) and hexadecyltrimethoxysilane (HDTMS, C_19_H_12_O_3_Si, Mw~346.62, 85%) as the combined modifiers were purchased from Sigma-Aldrich, China. Polydimethylsiloxanes (PDMS, (C_2_H_6_OSi)_n_, Sylgard 184 silicone elastomer) including its curing agent was purchased from Dow Corning Inc. The epoxy resin (two-component, clear, quick-setting) was purchased from the J-B Weld company, Sulphur Springs, TX, USA. Beech was cut into a series of small pieces of wood (length × width x height = 3 × 2 × 2) and used as the substrates.

### 2.2. Eexperimental Methodology

#### 2.2.1. Preparation of CNC@SiO_2_ Rod

**Figure 1:**
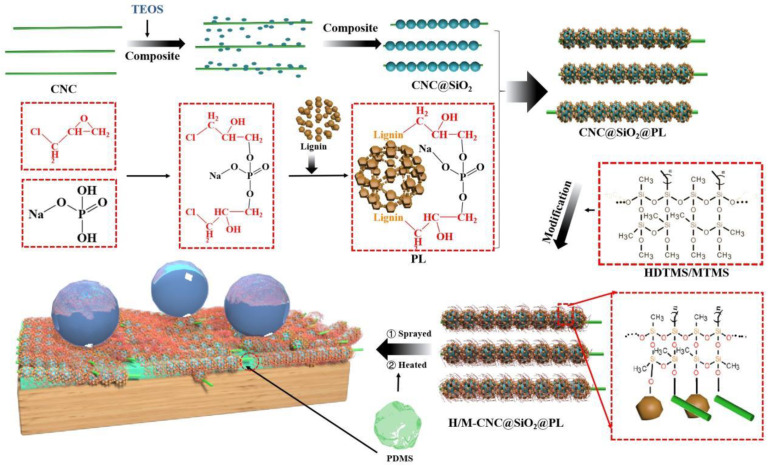
Preparation process demonstration and forming mechanism of CNC@SiO_2_@PL-based superhydrophobic wood, and possible chemical reactions during preparation.

The preparation process of CNC@SiO_2_ rod is shown in Figure 1. SiO_2_ grew on the surface of CNC as the axis under alkaline conditions to form CNC@SiO_2_ rods. Exactly as described in our previous study [6], CNC aqueous suspension was replaced with ethanol suspension by dialysis for three days, with the replacement of fresh ethanol every 12 h. The CNC ethanol suspension was diluted to a concentration of 1.5 wt%, mixed with ammonium hydroxide solution and TEOS with the volume ratio of 50:4:4, and stirred with 500 rad/min at 55 °C for ~2 h under sealing to a CNC@SiO_2_ rod mixture.

#### 2.2.2. Preparation of Phosphorylated Lignin (PL)

According to the reports [26], 35.8 g of Na_2_HPO_4_∙12H_2_O was dissolved in a flask with 50 g of distilled water at 50 °C and synthesized through adding 10 g of epichlorohydrin drop by drop, followed by keeping it at 85 °C for 5 h. Here, an intermediate (3-chloro-2-hydroxypropyl phosphate) was produced by the ring-open process and reaction with Na_2_HPO_4_∙12H_2_O of epichlorohydrin (Figure 1). After adjusting the pH to 11, lignin extracted by ethanol was added, and stirring continued for 5 h to get the phosphorylated lignin (marked as PL). PL was obtained by grafting phosphate groups onto the benzene rings of the lignin molecules under the substitution reaction between the intermediate and the active sites of lignin.

#### 2.2.3. Synthesis and Modification of CNC@SiO_2_@PL Rod

CNC@SiO_2_ water–ethanol suspension (water:ethanol = 6:1 *v*/*v*) was prepared and sonicated with a cell crushing apparatus at 500 W for 15 min. PL (mass ratio of PL:CNC@SiO_2_ = 1.3:1) was added to the CNC@SiO_2_ suspension and stirred for 30 min. Then, the pH of the system was adjusted to 3.5, and stirring continued for 30 min to get the CNC@SiO_2_@PL rods [27]. Both MTMS and HDTMS as the combined modifiers were added to the system, and stirring continued for ~4 h to achieve a sufficient reaction. Because both MTMS and HDTMS could mutually promote the hydrolysis in a certain concentration of ethanol solution, followed by dehydration and condensation to form a long hydrophobic carbon chain (as shown in Figure 1). Subsequently, the above mixture was placed in an oven at 40 °C for 1 h. Finally, the sample was collected by filtration with filter paper, followed by washing four times with distilled water and drying to obtain the hydrophobic CNC@SiO_2_@PL rods powder.

#### 2.2.4. Preparation of CNC@SiO_2_@PL Superhydrophobic Coating

The modified CNC@SiO_2_@PL mixture was redispersed in ethanol by magnetic stirring and adjusted to the concentration of 1.5 wt%, followed by adding PDMS (including its curing agent taking up PDMS of 1/10) as the adhesive at the half dosage to the CNC@SiO_2_@PL rods to obtain the hydrophobic paint. Subsequently, the hydrophobic paint was sprayed using a spray gun or spun by a rotary coating machine onto the substrate surface. Finally, the CNC@SiO_2_@PL@PDMS superhydrophobic coating was obtained after drying at 150° for 2 h.

To compare the influence of different adhesives on the mechanical strength of the CNC@SiO_2_@PL-based superhydrophobic coatings, epoxy resin was also used as the adhesive. First, the epoxy was treated on the wood surface. After 3~5 min, the CNC@SiO_2_@PL mixture was sprayed or rolled onto the surface of the epoxy layer, followed by standing for 1 h in the oven at 80 °C to obtain the CNC@SiO_2_@PL@epoxy superhydrophobic coating.

### 2.3. Characterization

Surface morphologies and microstructure of both different coatings and various rods were observed by scanning electron microscopy (SEM, FEI company, Hillsboro, OR, USA) and transmission electron microscopy (TEM, Zeiss, Oberkochen, Germany). The chemical compositions and elemental change of both lignin and CNC@SiO_2_@PL rods before and after modification were tested by Fourier transform infrared spectrometer (FTIR, PerkinElmer, Waltham, MA, USA) with the wavenumber range of 400~4000 cm^−1^ and 32 times of scanning) and X-ray photoelectron spectroscopy (XPS, PHI-5300 photoelectron spectrometer, PerkinElmer Instruments Co., Ltd., Waltham, USA). WCAs of different coatings were measured by a commercial contact angle meter (Shanghai Zhongchen JC2000D, China) with a rotatable sample platform, the sample was placed on the sample platform, and liquid droplets of 4–8 µL were dropped onto the sample surface through a controlled syringe, followed by adjusting the accuracy and measuring. The SAs were measured by tilting the sample platform until the liquid droplets started to roll, and the angle of rotation was taken as the SA. The measured result was taken at the average value of five random measurements.

The nanoindentation (NI) analysis of the coatings was conducted by a Berkovich indenter which was equipped with the iMicro nanoindenter (KLA corporation, San Francisco, USA) system. As described in the report [28], the NI sample prepared by the coating was uniformly spread on the substrate, and then the polished coating was uniformly spread on the substrate by an ultra-microtome (Leica MZ6, Germany) equipped with a diamond knife. NI mapping in a recently reported methodology [29] was utilized to analyze the static mechanical properties of the wood–adhesive interphase to obtain the corresponding cartographies of the mechanical performance. An array of 30 × 30 indents was conducted with a peak force of 200 μN in a covered area of 30 × 30 μm^2^. The reduced elastic modulus (*Er*) and hardness (*H*) can be calculated with the methodology developed by Oliver and Pharr in 1992 (Equations (1) and (2)). Since the 1970s, the relationship between stiffness, reduced elastic modulus, and the projected area had been obtained by analyzing the influence between indentation depth and load. However, the relationship was limited to the analysis of the results from cylindrical and conical indenters, not applicable to the results from the spring back of samples. Therefore, Oliver and Pharr developed a nanoindentation test method using a Berkovich indenter and taking into account the change in projected area due to sample rebound, which is the method used today. Please refer to [30] for details.

(1) $$Er=\frac{\sqrt{\pi }}{2\beta }\frac{S}{\sqrt{A}}$$

(2) $$H=\frac{P_{max}}{A}$$

*S* (mN/mm) is the initial unloading stiffness, and *β* refers to a correction factor correlated to the indenter’s geometry (*β* = 1.034 for a Berkovich indenter). *A* (µm^2^) stands for the projected contact area at peak load. *P_max_* (µN) corresponds to the peak load of indent.

### 2.4. Sandpaper Abrasion and UV Resistance Test

The sandpaper abrasion test was described in our previous study [6]. For this test, the coated side of the sample was placed on sandpaper (300 grit), and the loading with 50 g of weight was applied to the uncoated side of the sample. Then, the sample was moved forward by 10 cm under an action of the external force and returned to the original position in the same way. This was taken as one abrasion cycle. After each abrasion cycle, the WCAs were measured. The sandpaper abrasion test was not stopped until the average value of WCAs was less than 150°. The UV resistance test was conducted by placing the samples in a UV aging test chamber at a distance of 20 cm from the light source, and the WCAs and SAs were measured each 1 h (or 12 h) until the average value of WCAs was <150°. There were two kinds of UV light used, one with 15 W of power and a wavelength of 265 nm, and the other with 1000 W of power and a wavelength of 356 nm [31].

## 3. Results and Discussions

### 3.1. Surface Morphology

**Figure 2:**
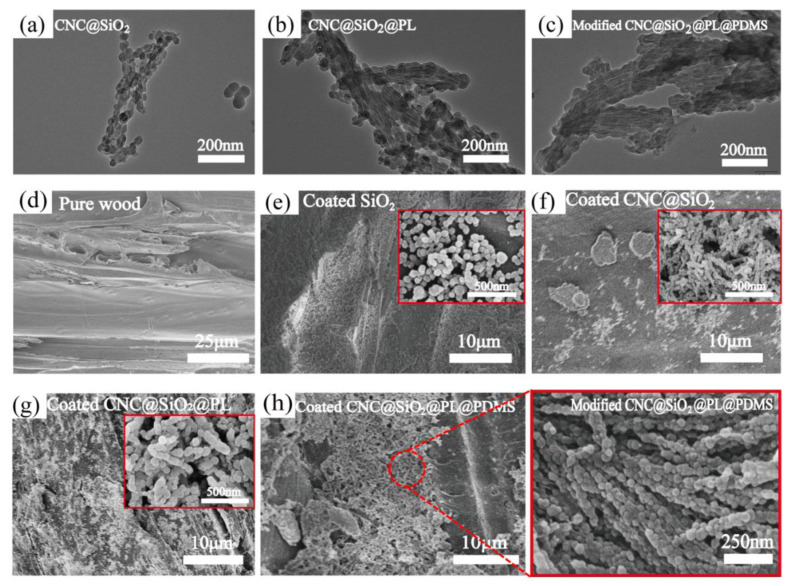
TEM images of (**a**) CNC@SiO_2_, (**b**) CNC@SiO_2_@PL, (**c**) modified CNC@SiO_2_@PL@PDMS coating; SEM images of (**d**) pure wood, (**e**) SiO_2_, (**f**) CNC@SiO_2_, (**g**) CNC@SiO_2_@PL, (**h**) CNC@SiO_2_@PL@PDMS coated woods. The illustrations in red box are the corresponding high magnification images.

As shown in the TEM images above, SiO_2_ nanoparticles grew in situ on the surface of CNCs to form the rod-bead type of CNC@SiO_2_ complex (Figure 2a), their diameter was about 20~50 nm. With the introduction of PL, it was obvious that the diameter of the CNC@SiO_2_@PL rods (Figure 2b), increased to about 30~80nm. The PL was precipitated onto the surface of the CNC@SiO_2_ rods to form a wrinkled surface, which was conducive to the promotion of the nanoscale structure. As shown in the SEM images, the wood surface was ragged due to uneven gullies formed from damaged conduits (Figure 2d), and SiO_2_ formed a coating on the wood by stacking with each other (Figure 2e). In the CNC@SiO_2_ coating (Figure 2f), its disorderly accumulation could build a better air nest due to the rod-like structure with a high length–diameter ratio, which was conducive to reducing the actual contact area between the water droplets and the coating surface, corresponding to the Cassie model [32]. This suggested that CNC@SiO_2_ rods were more suitable as the rough structure of superhydrophobic coatings, which also proved that the WCA of the CNC@SiO_2_ coating was higher than that of the pure SiO_2_ one (as shown in the section “*Wettability Analysis*”). In addition, CNC connecting SiO_2_ in series could improve the stability of the structure, which would be reflected in the following sandpaper abrasion test. Similarly, the CNC@SiO_2_@PL coating had better stability and superhydrophobicity than the SiO_2_@PL one (Figure 2g,h). The introduction of a small amount of PDMS had no significant effect on both the diameter of the CNC@SiO_2_@PL rods (Figure 2c) and the microstructure of the coating, which was mainly deposited on the bottom of the coating to provide adhesion.

### 3.2. Chemical Composition Analysis

**Figure 3:**
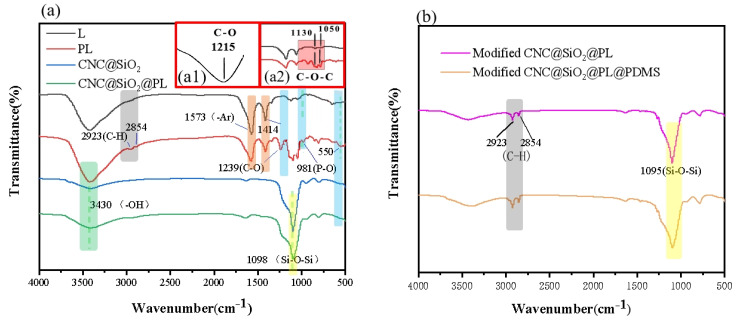
FTIR spectra of (**a**) lignin (L), PL and CNC@SiO_2_ and CNC@SiO_2_@PL rods and (**b**) the modified CNC@SiO_2_@PL and CNC@SiO_2_@PL@PDMS coatings; Local FTIR spectra of (**a1**) C-O bonds in L and (**a2**) C-O-Cbonds in L and PL.

In the system, the chemical composition was analyzed by FTIR (as shown in Figure 3a). According to the FTIR spectrum of lignin (marked L), before modification, the skeleton vibration of aromatic ring in the lignin appeared at 1573, 1414 cm^−1^ [33]. The absorption peak at 1215 cm^−1^ (Figure 3(a1)) was related to the stretching vibrations of the C–O bonds, and the absorption peaks (1130, 1050 cm^−1^) were mainly caused by the stretching vibrations of C–O–C bonds [34]. After modification, the phosphate groups were introduced to lignin to form PL by the substitution reaction of the –CH_2_Cl of the intermediate (3-chloro-2-hydroxypropyl phosphate) and the active sites of lignin. The stretching vibrations of the C–O bonds were moved to 1239 cm^−1^ due to the influence of the intermediate. The vibration peak of the phosphate group appeared at 550 cm^−1^ and 981 cm^−1^ [26,35], indicating that the phosphorylation modification of lignin was successful, which was consistent with the analysis in Figure 1. In the CNC@SiO_2_ rods, it could be seen that the absorption peak at 3430 cm^−1^ was caused by the rich –OH groups, and the absorption peak at 1098 cm^−1^ was caused by Si–O–Si bonds. The stretching vibrations of the C–H bonds were related to the peaks at 2923 and 2854 cm^−1^, and it could be seen that the peak strength of the CNC@SiO2@PL after hydrophobic modification was enhanced. This was caused by the large amounts of methyl from both MTMS and HDTMS. After combining with the PL, the CNC@SiO_2_@PL rods showed similar peaks as before the combination, indicating the successful attachment of PL. After further hydrophobic modification by MTMS and HDTMS and the introduced PDMS as the adhesive (Figure 3b), the absorption peaks at 1095 cm^−1^ resulting from the Si–O–Si bonds coincided with that of SiO_2_, it was difficult to judge the success of modification by FTIR.

**Figure 4:**
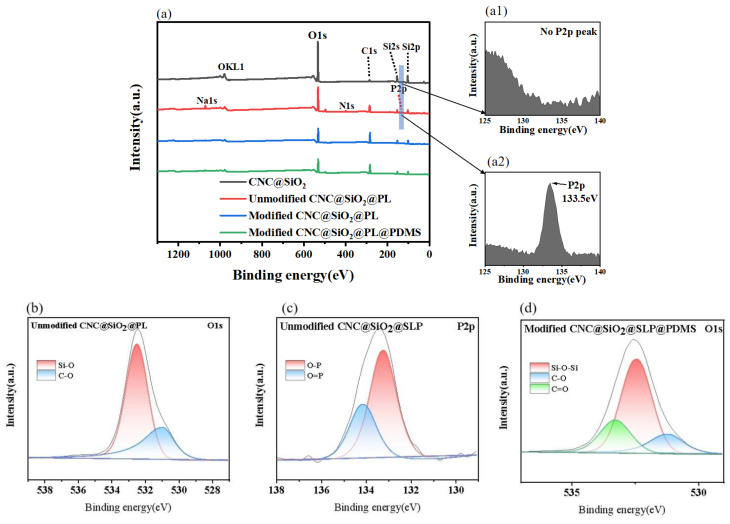
XPS spectra of (**a**) CNC@SiO_2_, CNC@SiO_2_@PL, modified CNC@SiO_2_@PL and CNC@SiO_2_@PL@PDMS coatings, (**b**) P2p spectra of unmodified CNC@SiO_2_@PL, (**c**,**d**) O1s spectra of unmodified CNC@SiO_2_@PL and CNC@SiO_2_@PL@PDMS coatings. Local enlarged XPS spectra of (**a1**, **a2**) elements P in CNC@SiO_2_ and CNC@SiO_2_@PL rods.

**Figure 5:**
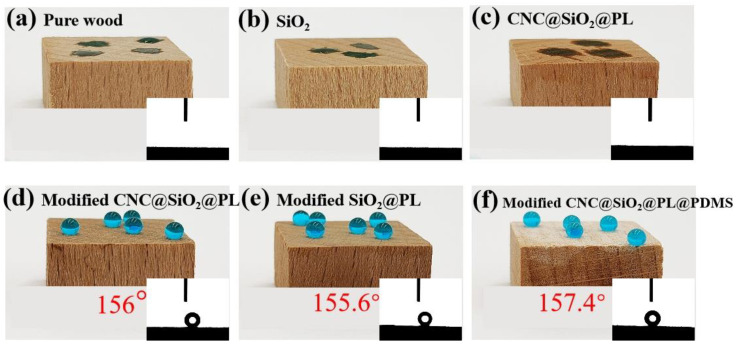
The droplets state on the surface of different samples: (**a**) pure wood, (**b**) SiO_2_, (**c**) CNC@SiO_2_@PL coatings; modified (**d**) CNC@SiO_2_@PL, (**e**) SiO_2_@PL, and (**f**) CNC@SiO_2_@PL@PDMS coatings.

Therefore, XPS was further used to verify the attachment of modifiers and to characterize the changes of surface elements of samples. The elements Si, O, and C could be clearly seen in the CNC@SiO_2_ rods (Figure 4a). In particular, element Si was obviously higher than element C because SiO_2_ was in the outermost layer and CNC was in the inner layer. When the PL was introduced, the peak intensity of element Si decreased, element P appeared, and the O–P bond existed (Figure 4b,c), directly indicating the successful covering of the PL. After further hydrophobic modification by MTMS, HDTMS, and the introduced PDMS, compared with that before modification, there were no new elements, because they both had the same chemistry bonds (Figure 4d). In Figure 5b, the Si–O bonds were from SiO_2_, whereas in Figure 5d, the Si–O–Si bonds were more commonly formed by the interaction of MTMS and HDTMS (as shown in Figure 1). The C=O bonds from CNC were detected in Figure 5d, but not in Figure 5b. It was possible that MTMS and HDTMS modifiers interact stronger with PL and SiO_2_, exposing CNC to the surface of the composite. Moreover, in the change of its element proportion, the element C further increased and the element Si reduced (as shown in Table 1), which could indirectly prove the successful attachment of the modifiers.

### 3.3. Wettability Analysis

As we all know, pure wood is mainly composed of cellulose, hemicellulose, and lignin, and it contains rich hydroxyl groups which easily absorb moisture (Figure 5a), resulting in poor dimensional stability and even the production of mildew [31]. This was also the reason that researchers were committed to the hydrophobic modification of wood. When the wood was treated only with the unmodified SiO_2_ nanoparticles, the sample showed stronger hydrophilicity (Figure 5b) than the pure wood due to their richer active hydroxyl groups. It is well known that lignin has fewer hydroxyl groups on its surface and its hydrophilicity is weak. After lignin was phosphorylated, the hydrophilicity of the resulting PL was enhanced. Therefore, the CNC@SiO_2_@PL-coated wood still showed good hydrophilicity (as shown in Figure 5c). After the introduced MTMS and HDTMS as the combined modifiers, the modified CNC@SiO_2_@PL and SiO_2_@PL coatings showed stable WCAs of 156° and 155.6°, and SAs of 6° and 7°, respectively (in Figure 5d,e). The variation of WCAs had no large change after the droplets were maintained for 1 h. This was due to the fact that the long hydrophobic chains formed from dehydration and condensation of the combined modifiers were attached to the surface of SiO_2_@PL and CNC@SiO_2_@PL rods, reducing their surface free energy. The modified SiO_2_@PL and CNC@SiO_2_@PL rods could be used to construct the superhydrophobic coatings by stacking them on top of each other.

It can be seen from the above that the presence of CNC had little influence on the wettability, which was mainly used to enhance the stability of the microscopic rough structure. PDMS, as a hydrophobic adhesive, was introduced to the system mainly to provide adhesion between the coating and the substrate. The WCA of CNC@SiO_2_@PL@PDMS reached 157.4° (Figure 5f) and the SA only 6°. As shown in Table 2, compared with other coatings, the WCA of CNC@SiO2@PL@PDMS was not inferior, proving that it had excellent superhydrophobic properties.

### 3.4. UV Resistance Test

**Figure 6:**
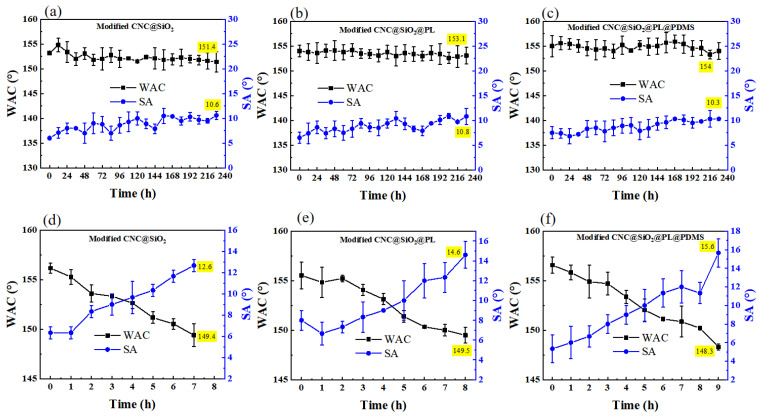
Change curves of WCAs and SAs on the modified CNC@SiO_2_, CNC@SiO_2_@PL, CNC@SiO_2_@PL@PDMS coatings with time of exposure to UV radiation of (**a**–**c**)15 W or (**d**–**f**) 1000 W.

When the prepared superhydrophobic coating was used outside, it would undoubtedly be exposed to the UV radiation from sunlight. Although most of the UV radiation from the sun is isolated by the atmosphere and reaches the ground only weakly, long-term accumulation does age the materials [39]. Therefore, the UV resistance test is one of the main indicators for the durability of superhydrophobic coatings. The outside UV environment was imitated using a low power UV lamp (15 W), as shown in Figure 6a–c. During UV irradiation for 10 days, the WCA of the samples had no significant change because the UV light was not strong enough to affect the coating during this time. To further estimate the UV resistance of the samples, the UV lamp was upgraded to an ultra-high power (1000 W). Under such strong radiation, the CNC@SiO_2_@PL coating showed a longer (7 h) UV resistance than the CNC@SiO_2_ coating (6 h), suggesting that the introduced PL had a certain influence on UV resistance. This was because lignin contained some functional groups such as benzene ring, carbonyl, double bond, and other conjugated structures, which were able to absorb certain UV rays [40]. The CNC@SiO_2_@PL@PDMS coating could endure 8 h before losing superhydrophobicity because PDMS also possessed a UV resistance effect [41], showing ultra-strong UV resistance (Figure 6d–f). It could be seen in Table 3, the CNC@SiO_2_@PL@PDMS coating showed better UV resistance than some similar work.

### 3.5. Abrasion Resistance Test Based on Nanoindentation Analysis

**Figure 7:**
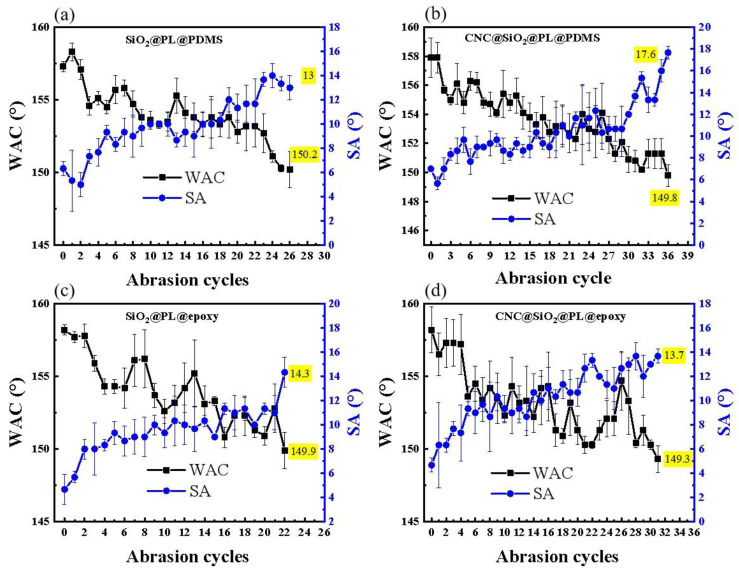
Change curves of WCAs and SAs on the modified (**a**) SiO_2_@PL@PDMS and (**b**) CNC@SiO_2_@PL@PDMS coatings, (**c**) SiO_2_@PL@epoxy and (**d**) CNC@SiO_2_@PL@epoxy coatings.

The methods to improve the mechanical properties of superhydrophobic coatings include sandpaper abrasion, sand free-fall impaction, cutting by a knife, etc. Among them, sandpaper abrasion is the one currently thought to be more able to imitate the destruction process of external forces, as well as comprehensive, convenient, and common methods [44]. As shown in Figure 7a, the SiO_2_@PL@PDMS coating could resist 27 abrasion cycles and show a gradual decrease of WCAs and increase of SAs during the sandpaper abrasion, resulting in the loss of superhydrophobic properties with the WCA of only 149.3°. The destruction by sandpaper led to the collapse of the rough structure of the coating and even the direct peeling of the coating, which would bring about the increased actual area between the water droplets and the coating surface and even the exposed hydrophilic area, thus increasing the adhesion of water droplets. That was the embodiment of the reducing WCAs and increasing SAs. It could be seen that the introduced CNC greatly improved the abrasion resistance of the superhydrophobic coating, and the abrasion cycles were up to 37 times in the CNC@SiO_2_@PL@PDMS coating. Compared with the SiO_2_@PL@PDMS coating, the CNC@SiO_2_@PL@PDMS coating benefited from the high crystallinity of CNC, which played a stable role in connecting the SiO_2_ particles together and made them difficult to erase in the abrasion process. When epoxy resin was taken as the adhesive, it could be also seen that the abrasion cycles of the coating with and without CNC were 22 and 31 times, respectively, which was a little worse than that of the coating with PDMS as the adhesive. This was determined by the properties of the adhesive. It was found that, in our previous research [31], the soft surface (PDMS) could be deformed under the external force during the abrasion process to offset part of the impact and have a certain degree of protection on the microstructure, but the hard surface (epoxy resin) could not.

The sandpaper abrasion test could visually show the mechanical strength of superhydrophobic coatings [44], but the result of this method was closely related to the grit size of the sandpaper. In the process of abrasion, the external force on the sample was asymmetrical, and the sandpaper incompletely contacted the coating surface. This might result in damage to only the local microstructure. This could not be mistaken for a loss of superhydrophobic properties. Therefore, to improve the shortcomings of the test methods above and to further determine the enhancement effect of the rod-bead rough structure constructed by the introduced CNC in the superhydrophobic coatings, nanomechanics analysis for the coatings was performed by NI mapping here. In the nanoindentation test, there was a small probe dotting the microstructure, and the stability degree of the microstructure was reflected according to the obtained nanoindentation images, so as to judge the mechanical strength of the coating.

**Figure 8:**
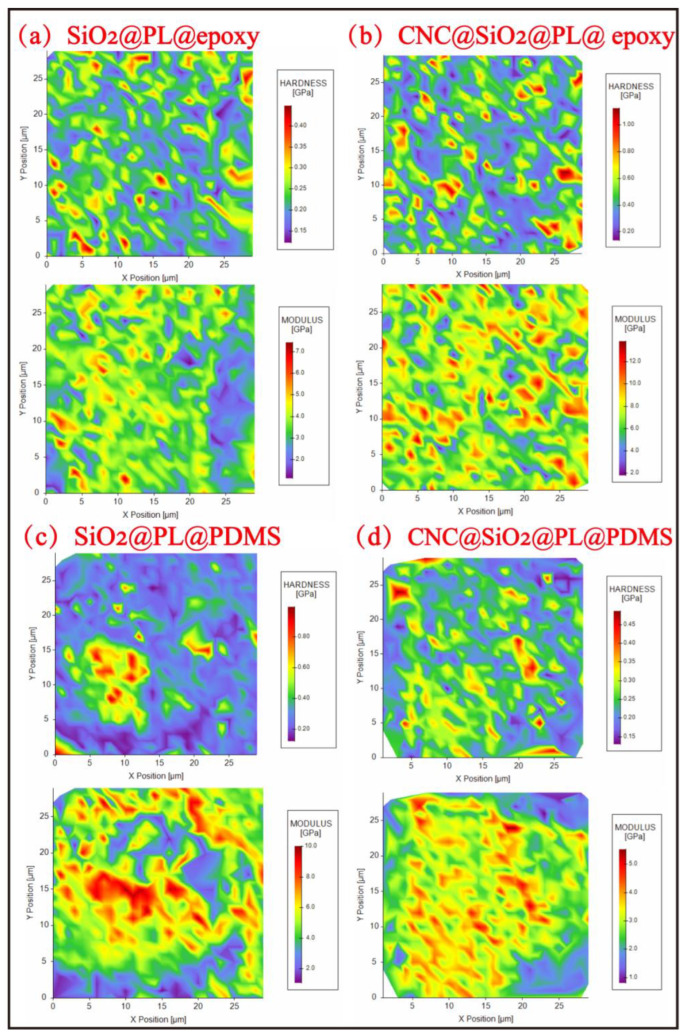
Nanoindentation analysis of different samples: (**a**) SiO_2_@PL@epoxy, (**b**) CNC@SiO_2_@PL epoxy, (**c**) SiO_2_@PL@PDMS and (**d**) CNC@SiO_2_@PL@PDMS coatings.

As shown in Figure 8, the *Er* and *H* cartographies of the coatings obtained by NI mapping demonstrate the significant role of CNC in elevating the mechanical performance of the coatings. When epoxy resin was used as the adhesive, the average *Er* and *H* for the coating without CNC were 5.53 and 0.34 GPa, respectively (Figure 8a). The obtained *Er* and *H* cartographies also indicated the uneven distribution of the mechanical performance, leading to the in-prior stress concentration when subjected to abrasion. The added CNC to the coating, however, clearly improved the mechanical performance, as illustrated in Figure 8b. It could be observed that the average *Er* and *H* elevated to 8.75 and 0.83 GPa, respectively. Moreover, compared with the one without CNC, the uniform distribution of the mechanical performance in the coating with CNC enabled better stress resistance.

Changing the adhesive to PDMS could also lead to the variation in the mechanical performance of the coatings. Yet the significant role of CNC in elevating the mechanical performance was still obvious. As displayed in Figure 8c, the average *Er* and *H* for the coating without CNC were 5.05 and 0.38 GPa, respectively. The existence of CNC promoted the formation of a 3D inner-network in the coating with PDMS, elevating the average *Er* and *H* to 8.3 and 0.47 GPa, respectively (Figure 8c,d). NI analysis demonstrated that the mechanical performance of the superhydrophobic coatings could be improved by CNC, which was beneficial to elevate the abrasion resistance by promoting the 3D inner-network of the coatings and forming a uniform distribution of mechanical performance. It could be also seen that the average *Er* and *H* of the coating with PDMS was a little lower than that of the one with epoxy resin. This was because PDMS as the adhesive had good elasticity. When the coating with PDMS was pressed into the coating by the NI probe during NI mapping, it could be able to produce good deformation and some energy dissipation occurred. Because of the energy dissipation, the coating was damaged less, which was more conducive to the protection of the rough structure of the coating with PDMS. But the epoxy resin was not, it was hard and its deformation was very small. Therefore, the rough structure on the surface of the coating with epoxy resin would be more seriously damaged. This was consistent with the result from analysis of the sandpaper abrasion process above.

## 4. Conclusions

In this study, CNC@SiO_2_@PL rods were successfully prepared by PL covering the surface of CNC@SiO_2_ rods formed by SiO_2_ in situ grown on CNC, and their hydrophobic properties were endowed by hydrophobic modifiers. A superhydrophobic coating with good abrasion resistance and UV resistance could be constructed using the modified CNC@SiO_2_@PL rods as the main materials and PDMS or epoxy resin as the adhesives. The WCA of the CNC@SiO_2_@PL coating could reach 157.4°, and the SA was only 6°. Due to the introduced CNC, SiO_2_ was connected in series by CNC to stabilize the microstructure. Therefore, compared with the pure SiO_2_@PL coating, the CNC@SiO_2_@PL coating was better in the abrasion resistance, resisting 37 abrasion cycles. PDMS also had a certain impact on the abrasion resistance because it could offset part of the impact force through deformation in the abrasion process. To further verify, nanoindentation tests were performed. The NI mapping results showed that the mechanical strength of the coating with CNC was better, which was consistent with the results of the macroscopic sandpaper abrasion test. In addition, the introduced PL was conducive to the improvement of UV resistance. Despite going under the super-high intensity of UV irradiation (1000 W), the superhydrophobic properties of the CNC@SiO_2_@PL coating remained for 8 h. Due to its good performance and simple preparation, the coating is expected to achieve real application in daily life.

## Figures and Tables

**Table 1:** Atomic ratio of elements in the CNC@SiO_2_ coating, CNC@SiO_2_@PL coatings before and after modification and modified CNC@SiO_2_@PL@PDMS coatings.

Samples	Atomic Percent (%)				
	C	N	Si	O	P
CNC@SiO_2_	6.04	0.69	28.32	64.85	-
CNC@SiO_2_@PL	28.88	3.57	13.8	51.76	2
Modified CNC@SiO_2_@PL	48.08	0.55	17.76	33.14	0.47
Modified CNC@SiO_2_@PL@PDMS	51.56	0.61	14.85	31.73	1.26

**Table 2:** Comparison of different superhydrophobic coatings for WCAs.

Superhydrophobic Coatings	WCA	Ref.
PDMS/SiO_2_ coating	153.6°	[36]
(PEI-H/SiO_2_)_3_@HDTMS coating	154°	[37]
PTMS-SiO_2_ coating	158.5°	[38]
CNC@SiO_2_@PL@PDMS coating	157.4°	This work

**Table 3:** Comparison of different superhydrophobic coatings for UV resistance.

Superhydrophobic Coatings	UV Resistance Time	UV Power	Ref.
PTMS-SiO_2_ coatings	10 h	8 W	[38]
The composite silane coating	200 h	60 W	[42]
SiO_2_ @ EP@ PDMS coating	2 h	1000 W	[43]
CNC@SiO_2_@PL@PDMS coating	8 (240) h	1000 (15) W	This work

## Data Availability

The data presented in this study is available on request from the corresponding author.
